# Early stage non-small cell lung cancer treated with pencil beam scanning particle therapy: retrospective analysis of early results on safety and efficacy

**DOI:** 10.1186/s13014-019-1216-1

**Published:** 2019-01-25

**Authors:** Jian Chen, Jiade J. Lu, Ningyi Ma, Jingfang Zhao, Chang Chen, Min Fan, Guoliang Jiang, Jingfang Mao

**Affiliations:** 1 Department of Radiation Oncology, Shanghai Engineering Research Center of Proton and Heavy Ion Radiation Therapy, Shanghai Proton and Heavy Ion Center, Shanghai, China; 2 Department of Medical Physics, Shanghai Engineering Research Center of Proton and Heavy Ion Radiation Therapy, Shanghai Proton and Heavy Ion Center, Fudan University Cancer Hospital, Fudan University, Shanghai, China; 3 Department of Thoracic Surgery, Shanghai Pulmonary Hospital, Tongji University School of Medicine, Shanghai, China; 4 Department of Radiation Oncology, Fudan University Shanghai Cancer Center, Fudan University, Shanghai, China; 5 Department of Radiation Oncology, Shanghai Engineering Research Center of Proton and Heavy Ion Radiation Therapy, Shanghai Proton and Heavy Ion Center, Fudan University Cancer Hospital, Fudan University, Shanghai, China

**Keywords:** Particle therapy, Proton, Carbon ion, Radiotherapy, Pencil beam scanning, Early stage non-small cell lung cancer

## Abstract

**Background:**

To evaluate the safety and efficacy of particle therapy (PT) using pencil beam scanning (PBS) technique for early stage non-small cell lung cancer (NSCLC).

**Methods:**

From 08/2014 to 03/2018, 31 consecutive patients with sum of the longest diameters of primary tumor and hilar lymph node < 5 cm, N0–1, M0 NSCLC treated with PT were retrospectively analyzed. Gating/active breathing control techniques were used to control tumor motion in 20 and 7 patients. PBS-based proton radiotherapy (PRT) or carbon ion radiotherapy (CIRT) plans were designed via Syngo® planning system. PRT, PRT + CIRT boost, and CIRT were used in 6, 6 and 19 patients, respectively. Prescriptions were categorized to 3 levels: 5–7.5 GyE * 8–10 Fx, 4–5 GyE * 15–16 Fx and 2.25–3.5 GyE * 20–31 Fx.

**Results:**

Thirty-one patients (20 males and 11 females) with a median age of 71 (50–80) years were enrolled with a median follow-up time of 12.1 (2.9–45.2) months. Fourteen were adenocarcinomas, 7 squamous cell carcinomas, 4 non-specified NSCLC and 6 had no histological diagnosis (4/6 had previous resected lung cancer). The median tumor size was 3.1 (1.1–4.7) cm. No grade 4–5 toxicities were observed. One patient experienced grade 3 (per the Common Terminology Criteria for Adverse Events version 4.03) radiation-induced lung injury (RILI) at 6.7 months from radiation started. Grade 2 acute toxicities included hematological toxicities (5 cases), RILI (2), plural pain (1) and dermatitis (1). Grade 2 late toxicities included RILI (3) and asymptomatic rib fracture (1). Three patients had progressed disease at 4.0~10.6 months after the initiation of PT. One experienced local failure with simultaneous distant failure and died of brain metastasis at 10.8 months; one developed regional and distant failure and died of lung infection at 8.7 months; the other experienced isolated distant failure only and his disease was well controlled after salvage systemic therapy. The estimated rates of progression-free survival, local control, cause-specific survival and overall survival at 1, 2 years were 85.5% and 85.5%, 95.2% and 95.2%, 95.0% and 95.0%, 90.7% and 90.7%, respectively.

**Conclusions:**

PBS-based PT appears safe and effective for early stage NSCLC. Further follow-up and investigation is warranted.

**Trial registration:**

ISRCTN, ISRCTN78973763. Registered 14 August 2018- Retrospectively registered, http://www.isrctn.com/ISRCTN78973763.

## Background

Lung cancer is the leading cause of cancer death worldwide. Approximately 80% of lung cancer patients are diagnosed with non-small cell lung cancer (NSCLC). Surgery is currently the standard therapy for early stage NSCLC. However, patients with severe underlying co-morbidities such as chronic obstructive pulmonary disease and cardiac insufficiency are not eligible for surgical procedures. Radiation therapy (RT), including stereotactic body radiotherapy (SBRT), has become the most favorable options for these patients [1].

With the physical dosimetrical advantage of particle beams and additional biological advantage of carbon ion beams compared with conventional radiation, particle therapy (PT) might be able to achieve better sparing of the normal tissue and even better tumor control and has therefore been used for the treatment of NSCLC patients with impaired pulmonary or heart function [2]. There are two major techniques implemented for PT: passive scattering (PS) and pencil-beam scanning (PBS). Theoretically, PBS can provide better dose distribution than PS in early stage NSCLC [3, 4]. However, PBS was relatively slowly applied to lung cancer worldwide because of the possible inaccuracy of dose distribution caused by the interplay effect, complicated tissue density variation of thorax area under PBS, respiratory motions, and range uncertainties of particle beams. Only several patients of early stage NSCLC were mentioned being treated by PBS-proton radiotherapy (PRT) in a previous study [5], no experience has been reported for carbon-ion radiotherapy (CIRT) using PBS technology for early stage NSCLC. After investigating how to deal with the interplay effect and delivery uncertainties for lung cancer, we successfully used PBS technique of PT to treat early stage NSCLC. In this retrospective study, we present our initial experience and clinical results at Shanghai Proton and Heavy Ion Center (SPHIC).

## Methods

### Patients and pretreatment evaluations

Patients with T1-2a, N0–1, M0 (American Joint Committee on Cancer staging manual version 7th) NSCLC and deemed medically inoperable or declined surgery were included in this study. The sum of the longest diameters of primary tumor and hilar lymph node was required to be less than 5 cm. All patients had pathologically confirmed NSCLC, or clinically diagnosed as NSCLC for the Fluorodeoxyglucose-Positron Emission Tomography/Computed Tomography (FDG-PET/CT) avid lung lesion and confirmed by two senior radiologists independently using computed tomography (CT) images. This study was conducted in accordance with the Declaration of Helsinki, and the protocol was approved by the Institutional Review Board (IRB) of the SPHIC (27/10/2017, 171020EXP-01). Written informed consents from all patients for using their data were obtained for this study.

Pretreatment evaluation for all patients included a complete disease history and physical examination, complete blood count, serum electrolytes, renal and liver function tests, electrocardiogram, pulmonary function tests and a mandatory FDG-PET/CT scan for clinical staging.

### Particle therapy (PT)

#### Patient immobilization

The patients were positioned in supine or prone position based on the location of the radiation targets using a vacuum bag. A thermoplastic mask was used to fix the position and restrict the breath motion for patients using free breathing or gating.

#### Tumor motion management, simulation CT and contouring

All patients were evaluated for tumor motion under fluoroscopy before simulation. If the tumor motion in all the directions was less than 5 mm, the patient would be treated under free breathing (FB); if the motion exceeded 5 mm, a breath control technique, either active breathing control (ABC) or respiratory gating, was required to mitigate the residual motion to < 5 mm during treatment.

The simulation CT scanning started from the angle of mandible to adrenal glands to include tumor lesions, entire lungs, whole neck and all the organs/tissues by which the beams probably pass. CT scanning was performed under the same respiratory mode as that would be used for PT treatment. All CTs for planning were plain CT, and a contrast CT afterwards was scanned for image fusion only when needed.

For patients to be treated under FB or ABC, a plain simulation CT was scanned under the same respiratory mode. The gross tumor volume (GTV) was delineated on the plain CT.

For patients using gating, a 4-dimensional (4D) simulation CT was performed. Ten phases of a whole respiratory cycle were reconstructed. The gating window (respiratory phase time when beam is on, usually around the end of exhale) was selected to restrict the tumor motion to < 5 mm. The GTV was delineated on one phase of them (usually the end of exhale), then propagated to all other phases using deformable registration and confirmed by physicians. The GTVs generated on each phase were combined to form the internal gross tumor volume (iGTV).

The clinical target volume (CTV) was defined as a 0.6–0.8 cm expansion from GTV/iGTV. Range uncertainties and set-up errors were counted when creating the planning target volume (PTV). In most instances, it was CTV plus a 0.5–0.7 cm margin in lateral and a 0.7–1.5 cm margin along beam direction.

#### Prescriptions

The dose of PT is defined as equivalent dose to Gy of photon (GyE). Three radiation beam sources were used for this group of patients: PRT only, PRT + CIRT boost, or CIRT only. The selection of radiation sources or their combination depended on the clinical trials patients involved. For patients who did not participate in clinical trials, patients were treated according to our IRB approved institutional treatment protocols. From March 2016 ~ July 2017, 3 prospective phase I/II clinical trials approved by hospital IRB were started to enroll patients with stage I NSCLC. According to the distance from the primary lesion to critical organs at risk (OARs), patients were categorized to have peripheral, median, and central lesions. Overall, dose and fractions used included 5–7.5 GyE * 8–10 Fx, 4–5 GyE * 15–16 Fx and 2.25–3.5 GyE * 20–31 Fx.

At least 99% of GTV covered by 99% of the prescription dose, 99% of CTV covered by 95% of the prescription dose, and 90% of PTV covered by 90% of the prescription dose were required. All treatment plans aimed to meet stringent guidelines for protection of OARs, including lungs, spinal cord, esophagus, trachea, proximal bronchi, and heart/big vessels. The normal tissue dose-volume constraints for conventionally fractionated and ablative radiotherapy of the National Comprehensive Cancer Network guideline as well as published results from M.D. Anderson Cancer Center (MDACC) and the University of Florida Proton Therapy Institution were considered to set up the dose constraints for our patients according to fraction size [6–10].

#### Particle therapy planning

**Fig. 1:**
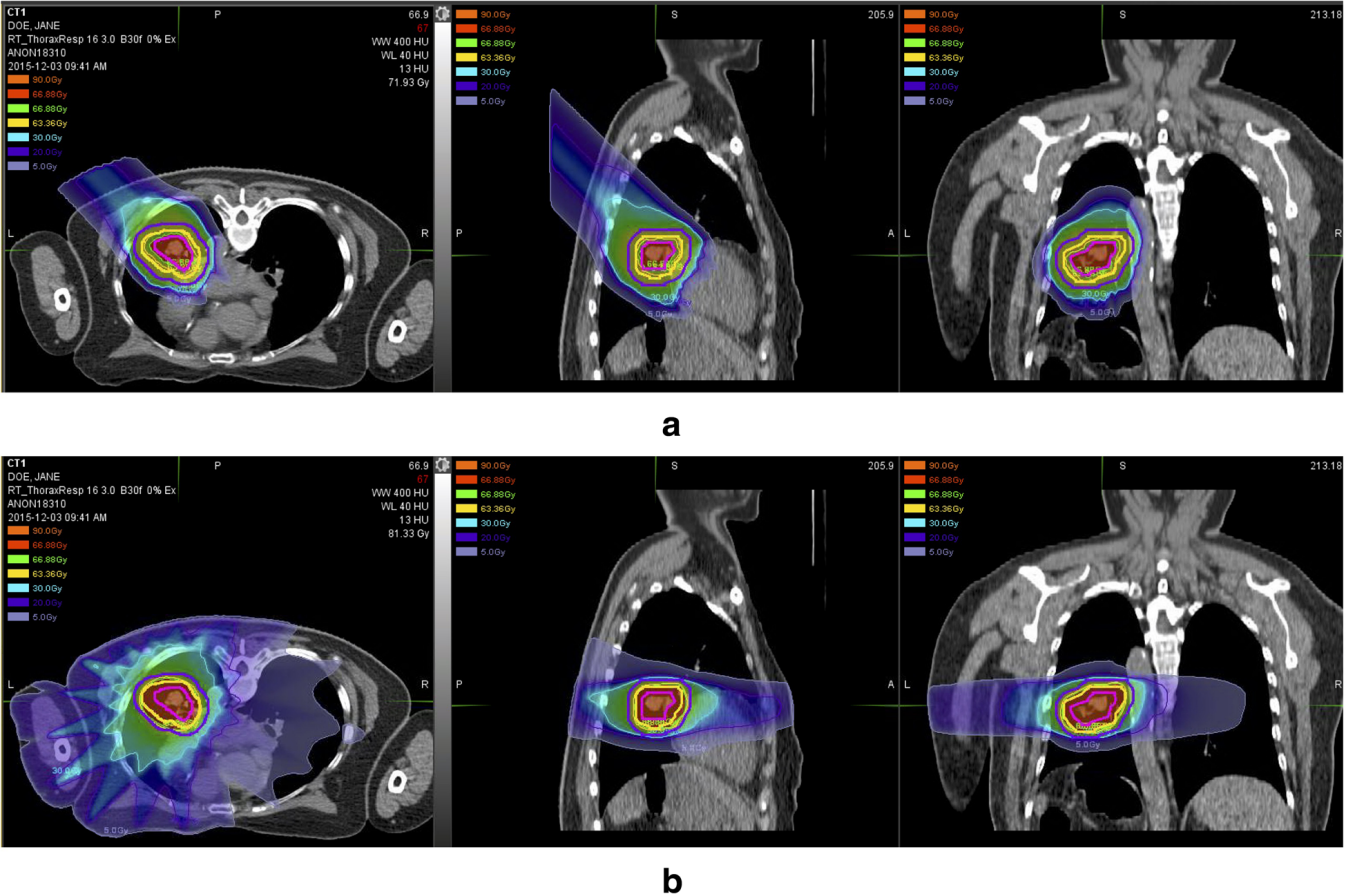
Typical dose distribution of CIRT (pencial beam scannning) (**a**) vs. X-ray based radiation therapy (RapidARC®) (**b**). CIRT significantly reduced the doses to the contralateral lung, heart, spinal cord, and the volume of low-dose area as compared to X-ray based arc therapy

The Siemens Syngo® treatment planning system was used for all patients’ PT planning. PRT (beam energy 30~250 MeV) and CIRT (beam energy 85~430 MeV) plans were designed by using 2 to 4 beams with PBS technique. Single beam optimization (SBO) or intensity modulated particle therapy (IMPT) method was decided to be used in treatment by evaluating delivery uncertainties and doses to critical organs during planning. Without the feature of robustness evaluation in the Syngo® system, several steps were applied to reduce the uncertainties. First, similar to the experience of MDACC [5], the threshold of 5 mm was selected for motion control in our center. Based on the physical experiment on Alderson phantom conducted in our center, the deviation of dose distribution was found to be acceptable (< 5%) when respiratory movement was 4.4 mm or less. Second, iGTV of the plan was contoured to include all tumor trajectory in 10 phases, and the density of the intersection of iGTV and GTV will be overridden to a similar density of the primary lesion before planning. Third, when planning, physicists will avoid the beam angles passing through the heart and great vessels and keep the beam path less inhomogeneous. Forth, for hypo-fractionation plans (fraction dose ≥6 GyE), strategies of repeated SBO which is mimicking the volumetric rescanning [11], and specific beam PTV expansion were typically used to increse the plan robustness. IMPT was only used for lesions with minor motions, or large volumes and irregular shapes. Fifth, large sport size (full width at half maximum, FWHM = 10 mm) and decreased iso-energy slice spacing (Grid resolution = 2 mm) were used to increase target dose heterogeneities [12]. Sixth, 4D plan recalculation and accumulated dose also have been used for hypo-fractionation plans to evaluate dose distribution. Last but not least, before the first treatment and every week during treatment, simulation CT and plan recalculation on the latest CT will be conducted for every patient, to check the dose distribution, and replanning was demanded when recalculation revealed poor coverage of targets or overdosing to the OARs. Two orthogonal X-ray images were taken to verify and registrate patient position according to bone structures before daily irradiation. The set-up error of ≤3 mm before the treatment was allowed. Typical cases of CIRT are shown in Fig. 1.

### Follow up and evaluation

All patients were evaluated weekly for treatment-induced toxicities and disease response/progression during their treatment. After the completion of PT, all patients are required to be evaluated according to our institutional follow-up protocol for lung cancer at 3 months after the 1st day of PT, every 3–4 months within the first 2 years, every 6 months between year 3 and 5, and annually thereafter. Treatment-induced side effects were scored using the Common Terminology Criteria for Adverse Events (CTCAE) version 4.03, for events observed after the first dose of irradiation. Toxicities occurred 90 or more days after the completion of PT were defined as late toxicities. The Response Evaluation Criteria in Solid Tumors (RECIST) version 1.1 was used for tumor response evaluation.

### Statistical analyses

The overall survival (OS) time was calculated from the date of pathological diagnosis of the primary disease or radiological diagnosis of disease for patients without pathological confirmation until death or the date of the last follow-up. The time to local, regional, or distant failure was calculated from the date of the first fraction of PT until documented first considered treatment failure. Local control (LC) and various survival rates were calculated using the Kaplan-Meier method. All analyses were performed in SPSS statistics version 21 (Armonk, NY; USA).

## Results

### Patient characteristics

Between August 2014 and March 2018, 31 consecutive and non-selected patients with NSCLC met the inclusion criteria were included in this study. Histological diagnoses were obtained in 25 patients. Biopsy were either declined or contraindicated for the remained 6 patients, including 4 diagnosed as the second primary lung cancer with previous resected primary NSCLC in other locations. All patients had stage I-IIa disease. Eight patients refused surgery, and 23 patients were medically inoperable (including chronic obstructive pulmonary, interstitial lung disease, cardiovascular disease, etc.). Patient characteristics are detailed in Table 1.

**Table 1:** Characteristics of patients

	Number of Patients
Age (year)	71 (50~80)
Gender	
Male	20
Female	11
ECOG score	
0/1	19/12
Pathology	
SCC/Adno/ Unclassified/None	7/14/4/6
Primary tumor size, median (range)	3.1 (1.1~4.7) cm
Primary tumor volume, median (range)	19.39 (0.97~56.23) cm^3^
Stage (AJCC staging v7) I/IIa	21/10
T1aN0M0	4
T1bN0M0	7
T2aN0M0	10
T1aN1M0	1
T1bN1M0	4
T2aN1M0	5
Tumor location	
Left/right lung	9/22
Upper/middle/lower lobe	19/2/10
Peripheral/median/central type-SPHIC institutional definition	3/15/13
Smoking history (packyears), median (range)	15 (0~150)
Refusal of surgery	8
Medically inoperable	23

Abbreviations: *ECOG* Eastern Cooperative Oncology Group, *SCC* Squamous cell carcinoma, *Adno* Adenocarcinoma, *GTV* Gross tumor volume, *AJCC* American Joint Committee on Cancer, *SPHIC* Shanghai Proton and Heavy Ion Center

### Particle therapy and chemotherapy

#### Particle therapy

The breath control techniques, beam types and doses of PT used are detailed in Table 2. Most patients (27/31) were treated with breath control techniques. Prescriptions of biological effective dose (BED, α/β = 10) < 100 GyE (using relatively low fraction dose) mostly prescribed to patients with N1 disease. The mean values of doses to thoracic OARs of all patients were listed in Table 3.

**Table 2:** Characteristics of treatments

	Number of Patients
Breath control techniques	
Gating	20
Active breathing control	7
Free Breathing	4
Prescriptions	
5–7.5 GyE X 8–10 Fx	12
4–5 GyE X 15–16 Fx	3
2.25–3.5 GyE X 22–31 Fx	16
BED_10_ (GyE), median (range)	96 (75–119)
Radiation techniques/sources	
PRT only	6
PRT + CIRT boost	6
CIRT only	19^a^
Chemotherapy	6
1 cycle (neoadjuvant of pemetrexed+ cisplatin)	1
3 cycles (concurrent of Taxol+ cisplatin)	1
4 cycles (neoadjuvant of etoposide+ cisplatin)	1
4 cycles (neoadjuvant of pemetrexed X 3+ docetaxel X 1)	1
5 cycles (neoadjuvant of gemcitabine+ cisplatin X 3 + concurrent of Taxol+ cisplatin X 2)	1
6 cycles (neoadjuvant of gemcitabine+ cisplatin)	1

*Abbreviations:BED*_*10*_ Biological effective dose (α/β = 10), *PRT* Proton radiation therapy, *CIRT* Carbon ion radiotherapy

^a^Included one patient discontinued X-ray based Intensity Modulated Radiation Therapy (IMRT) after 2 fractions due to pneumonia, and received CIRT for 69 GyE/23 fractions

**Table 3:** The mean values of doses to thoracic OARs of all patients (GyE)

	MLD	Lungs V5 (%)	Lungs V20 (%)	MHD	MED	Dmax of Eso	Dmax of SC	Dmax of MBT
Mean	6.31	18.42	11.13	1.88	4.24	29.28	13.65	46.01
SD	2.80	7.45	5.77	2.56	5.58	25.65	14.12	29.06

*Abbreviations: MLD* Mean lung dose, *V5/V20* The volume of lung parenchyma that received 5/20 Gy or more, *MHD* Mean heart dose, *MED* Mean esophageal dose, *Dmax* Maximum dose, *Eso* Esophagus, *SC* Spinal cord, *MBT* Main bronchus tree, *SD* Standard deviation

#### Chemotherapy

Chemotherapy was recommended to 16 patients with larger tumor (> 4 cm in diameter) and/or N1 disease; however, only 6 received some form of chemotherapy due to co-morbidities or patients’ refusal. Chemotherapy regimens and schedules were detailed in Table 2.

### Survival and disease control

Till the last follow up on July 16th, 2018, the median follow-up time was 12.1 (range 2.9–45.2) months for the entire cohort of the patients. Three patients had progressed disease and 2 out of them died during follow-up. One experienced simultaneous local and distant failure at 7.3 months, then died of brain metastasis at 10.8 months; one suffered simultaneous regional (lymph node out of the radiation field) and distant failure at 4 months, and died of fungal pneumonia at 8.7 months after the completion of CIRT. The third patient developed distant metastasis only at 10.6 months, and the disease was well controlled after salvage chemotherapy and immunotherapy. In addition, one patient diagnosed as T1N1M0 disease had a single bone lesion considered as benign before PT. The bone lesion was proved as metastasis after the completion of PT. He received palliative photon RT to it and systemic chemotherapy. Both the thoracic and bone lesions remained stable at his last follow-up.

Two patients developed a second primary lung cancer after 25.3 and 31.5 months of particle therapy. The new lesion of one patient occurred from a chronic lung lesion which already existed near to the original primary lesion before PT and was not included in the proton irradiation area; that of the other was close to PT irradiation area but with different pathological type. In addition, two patients were diagnosed with rectal and gastric cancer, respectively, at 1 year and 3 months after PT and received surgery or palliative treatment.

**Fig. 2:**
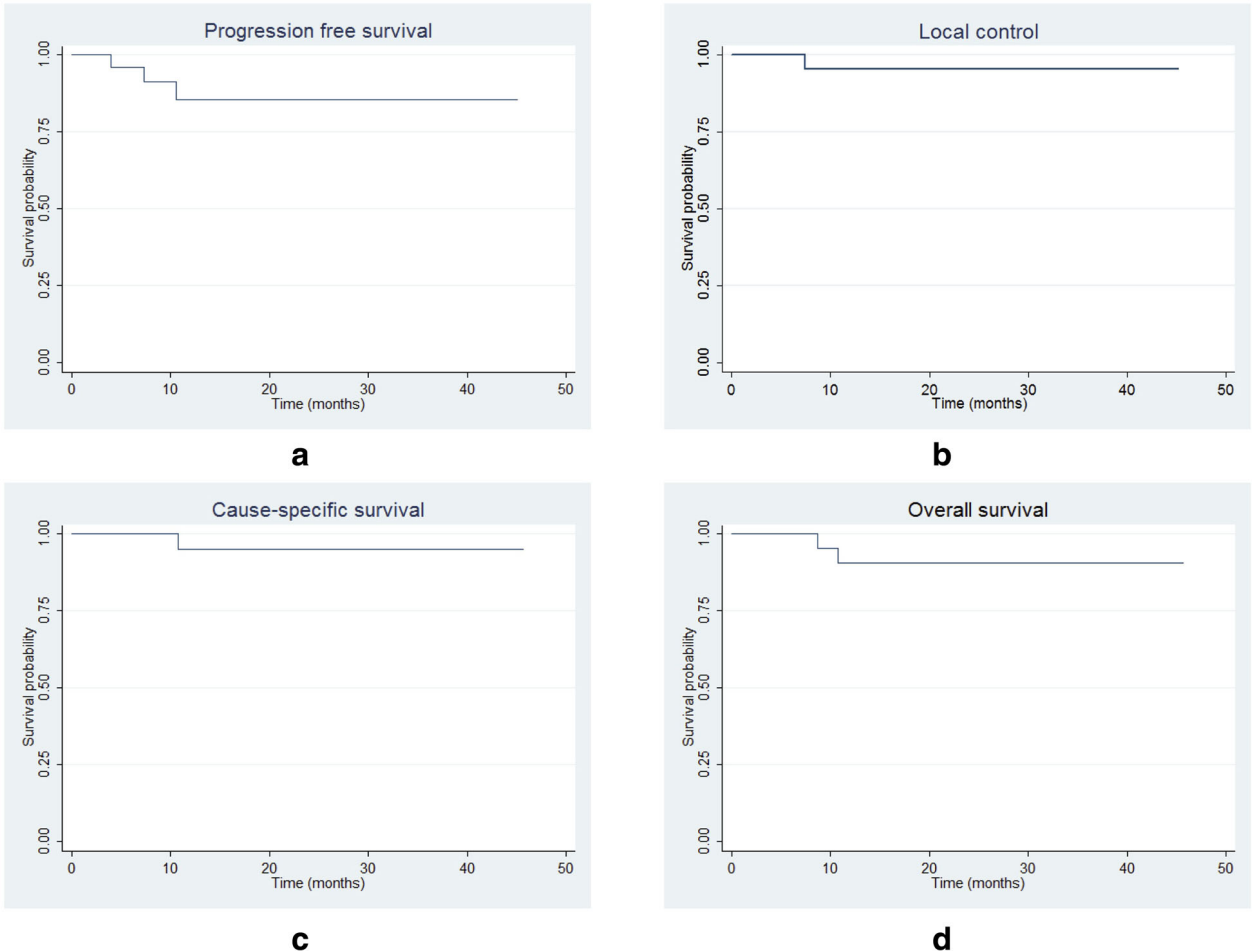
The estimated curves of local control and various survival rates. Panel **a** shows that of progression-free survival (PFS), panel **b** local control (LC), panel **c** cause-specific survival (CSS), and panel **d** overall survival (OS)

The estimated rates of progression-free survival (PFS), local control (LC), cause-specific survival (CSS) and (OS) at 1, 2 years were 85.5% and 85.5%, 95.2% and 95.2%, 95.0% and 95.0%, 90.7% and 90.7%, respectively (Fig. 2).

### Acute and late toxicities

Grade 2 acute toxicities included hematological toxicities (5 cases), pneumonitis (2), plural pain (1) and dermatitis (1). Grade 2 late toxicities included radiation-induced lung injury (RILI) (3 cases) and asymptomatic rib fracture (1). One patient experienced grade 3 RILI at 6.7 months from the initiation of PT and recovered to grade 1 after hospitalization. No other toxicities of grade ≥ 3 were observed (Tables 4 and 5).

**Table 4:** Acute toxicities of the entire cohort

Acute toxicities	Grade		
	1	2	3~5
Dermatitis	3	1	0
Esophagitis	3	0	0
Pneumonitis	3	2	0
Plural pain	1	1	0
Cough	2	0	0
Leukocytopenia	4	3	0
Neutropenia	4	2	0
Anemia	3	0	0
Thrombocytopenia	1	0	0

**Table 5:** Late toxicities of the entire cohort

Late toxicities	Grade		
	1	2	3~5
RILI	11	3	1
Rib fracture	0	1	0
Pleural effusion	2	0	0

*Abbreviation: RILI* Radiation-induced lung injury

## Discussion

In the current study, by using proper breath control technique and other physical measurements to mitigate the interplay effect and delivery uncertainty, we successfully used PBS technique of PRT or CIRT to treat a group of 31 consecutive and non-selected patients with early stage NSCLC. After a median follow-up time of 12.1 months, we achieved a CSS and LC rates of 95.0% and 95.2% at 2 years with mild acute and late PT-related toxicities in a cohort of patients with stage I and IIa NSCLC (~ 1/3 of patients with stage IIa disease) and a median age of 71. Furthermore, approximate 80% of the patients had medically inoperable disease and most patients had centrally located lesions (29/31) per RTOG (Radiation Therapy Oncology Group) 0813 protocol [13]. Only 1 patient developed local recurrence due to, at least in part, the relatively low BED_10_ (77.2 GyE) because of poor baseline pulmonary functions. All 3 patients who developed distant metastases had larger tumor size (diameter > 3 cm) with insufficient chemotherapy. PT-induced toxicities were mild and relieved quickly after proper medical treatment. Only 1 patient who had infectious pneumonia just before the initiation of PT experienced grade 3 RILI and had recovered to grade 1 after hospitalization. PBS-based PT appeared feasible, safe, and effective for early stage NSCLC in a short-term observation.

Radiation therapy is the most important alternative to surgery for unresectable or medically inoperable early stage NSCLC. Radiation therapy with conventional fractionation has generally resulted in inferior LC and OS when compared with surgery. For medically inoperable stage I or II NSCLC, the OS and local failure rates were 24~40% and 25~70% at 2 years, respectively [14]. SBRT achieved better tumor control with more precisely delivered doses to the primary lung lesions with better protection of normal tissues, as compared to conventional RT. Reports [15–17] demonstrated that clinical outcomes after SBRT could be compared to those of surgery with 2,5-year local control rates of 67.9–97%, 73–91.9% and 2,5-year overall survival rates of 48–90%, 43–55.7%. However, for central/ultra-central type lesions, SBRT is of high risk for severe toxicities. Grade 3 toxicities could reach as high as 38% and fatal pulmonary hemorrhage (grade 5) could be even up to 15% [18, 19]. In this study, only 1 patient developed grade 3 RILI among 7/31 patients with ultra-central type lesions.

Due to their physical characteristics, particle beams provide superior dose localization for deep-situated tumor. The beams penetrate body and abruptly stop to form a Bragg peak where most of the energy was released. Dosimetric studies have shown that PT not only provides comparable or even better coverage of the PTV relative to standard SBRT plans, but also can provide better protection for OARs such as lungs, heart, spinal cord and esophagus [20–22]. In addition to the physical dosimetrical advantages, carbon-ion is also featured with high liner energy transfer (LET) thus higher relative biological effect (RBE) and lower oxygen enhancement ratio compared to photon. The reported 2-year LC and OS rates of proton therapy reached about 80–97% and 74–97.8%, respectively, which are comparable to SBRT with a generally lower incidence of severe toxicities [23–25]. For CIRT, Japanese studies showed a 3-year LC and OS of 82% and 75%, 5-year LC of 90–94.7% and OS of 45–50% without any grade ≥ 3 pulmonary toxicities [26–28].

PBS technology can provide more conformal dose distribution to targets and spare the normal tissues better theoretically, and is considered the future trend of technology development for PT [3, 4]. However, all studies published so far on early stage NSCLC’s particle therapy used passive scattering technique except several patient cases [5]. The major concerns for using PBS technology in the management of intra-thoracic lesion(s) are inaccuracy of dose distribution caused by inter-and intra-fractional organ motions (interplay effect), significant difference of CT densities among multiple organs and the stopping power uncertainty of particle beam which is more obvious when tumor size or anatomy changes during the treatment [11]. In our center, we followed up almost all the requirements in the consensus by Chang et al. [11] except for those we could not accomplish because of the feature of Syngo® system. However, we took several steps to increase our plans’ robustness as metioned in the ‘methods’ part. This paper was intended to present the promising preliminary results of our study.

PBS technique used for lung cancer was controversial with the concerns of the interplay effect. To the best of our knowledge, this is the first report in early stage NSCLC using PBS technique PRT and CIRT for the entire patient cohort. With the breath control techniques and other physical planning methods used, the 2-year CSS and LC rates reached 95.0% and 95.2% for whole cohort with mild toxicities. Considering the relatively unfavorable disease characteristics in this cohort, our results in terms of disease control and OS are very promising compared to previous reports using SBRT, PS-PT or PS-CIRT. Higher BED should be administered under a prospective dose escalation study to reduce local failure. Better results could be expected with optimal systemic therapy such as target therapy and/or immunotherapy.

There were some limitations of this study. First, although we collected the clinical data prospectively, it is still a retrospective study with a small number of patients. Second, the particle resources (proton or carbon ion) and prescriptions used for these 31 patients varied, thus complicated the statistical analyses in a study with a small sample size. Prospective phase I/II clinical trials per different tumor locations have been carrying out for dose escalation of pure carbon ion beam in patients with early stage NSCLC. Further investigation and follow-up of particle therapy using PBS technique are warranted to better define its role in early stage NSCLC.

## Conclusions

To the best of our knowledge, this is the first report in early stage NSCLC using proton and/or carbon ion beams with pencil beam scanning technique. Within a short-time follow-up, PBS-based PT achieved safe and effective results for early stage NSCLC. Further follow-up and investigation is warranted.

## Abbreviations

4-D: 4-dimensional
ABC: Active breathing control
Adno: Adenocarcinoma
AJCC: American Joint Committee on Cancer
BED: Biological effective dose
BED_10_: Biological effective dose (α/β = 10)
CIRT: Carbon ion radiotherapy
CSS: Cause-specific survival
CT: Computed tomography
CTCAE: Common Terminology Criteria for Adverse Events
CTV: Clinical target volume
ECOG: Eastern Cooperative Oncology Group
FB: Free breathing
FDG: Fluorodeoxyglucose
FWHM: Full width at half maximum
GTV: Gross tumor volume
GyE: Equivalent dose to Gy of photon
iGTV: Internal gross tumor volume
IMPT: Intensity modulated particle therapy
IMRT: Intensity modulated radiation therapy
IRB: Institutional Review Board
LC: Local control
LET: Liner energy transfer
MDACC: M.D. Anderson Cancer Center
NSCLC: Non-small cell lung cancer
OARs: Organs at risk
OS: Overall survival
PBS: Pencil beam scanning
PET/CT: Positron Emission Tomography/ Computed Tomography
PFS: Progression-free survival
PRT: Proton radiotherapy
PS: Passive scattering
PT: Particle therapy
PTV: Planning target volume
RBE: Relative biological effect
RECIST: Response Evaluation Criteria in Solid Tumors
RILI: Radiation-induced lung injury
RT: Radiation therapy
RTOG: Radiation Therapy Oncology Group
SBO: Single beam optimization
SBRT: Stereotactic body radiotherapy
SCC: Squamous cell carcinoma
SPHIC: Shanghai Proton and Heavy Ion Center
